# Eating Disorder vs Addison’s Disease: A Case Report and Review of the Published Case Reports

**DOI:** 10.62641/aep.v53i3.1840

**Published:** 2025-05-05

**Authors:** Ruben Touzon

**Affiliations:** ^1^Eating Disorders Unit, Department of Psychiatry, Basurto University Hospital, Osakidetza-Servicio Vasco de Salud, 48013 Bilbao, Spain

**Keywords:** eating disorder, Addison’s disease, case report

## Abstract

**Aim:**

This report presents the case of a 56-year-old female, initially diagnosed with an eating disorder, who was ultimately found to suffer from Addison’s disease. The aim is to highlight the differences between these two conditions to prevent future misdiagnoses.

**Case Presentation:**

The patient was admitted to the hospital under the care of the Internal Medicine Department due to an electrolyte imbalance. Following consultations with Psychiatry and Endocrinology, further evaluations led to the diagnosis of adrenal insufficiency. This case prompted a review of the literature on this topic. A comprehensive PubMed search identified nine published case reports of patients with adrenocortical insufficiency who were initially misdiagnosed with an eating disorder.

**Result:**

The data from these 10 cases, including the present one, were analyzed in terms of age, sex, diagnostic delay, symptoms, laboratory abnormalities, and clinical outcomes.

**Conclusions:**

Addison’s disease is a rare and potentially fatal condition whose symptoms can sometimes be mistaken for those of an eating disorder. It is crucial for psychiatrists and other specialists to consider this differential diagnosis in similar clinical presentations.

## Introduction

Thomas Addison [1, 2] first described Addison’s disease (AD), also known as adrenal insufficiency or suprarenal insufficiency, in 1855. AD is a chronic disorder of the supra-renal cortex resulting in low secretion of glucocorticoids and mineralocorticoids. The first cause of AD in developed countries is autoimmune, with many patients also presenting with autoimmune comorbidities such as Type 1 Diabetes Mellitus or autoimmune hypothyroidism. When this occurs, the condition is called autoimmune polyendocrine syndrome (APS), which has been associated with rheumatological diseases like rheumatoid arthritis [3].

Although AD is rare, its prevalence is estimated to be between 93–140 per million people, with an annual incidence of 4.7–6.2 per million people in Western populations [4, 5].

The symptoms of AD can either progress slowly over months or years or manifest in the form of acute adrenal crises, which can lead to hypovolemic shock. The insidious onset of symptoms and their nonspecificity (asthenia, lack of appetite, nausea and vomiting) often cause diagnostic delays. A hallmark symptom that may aid in diagnosis is skin hyperpigmentation, although it is not present in all cases. Electrolyte imbalances (often related to mineralocorticoid deficiency) can provide diagnostic clues, including hyponatremia, hyperkalemia, and metabolic acidosis (see Table 1 (Ref. [6]).

**Table 1.** **Key similarities and differences between anorexia nervosa and primary Addison’s disease (Adapted from Nicholls *et al*. [6])**.

Anorexia nervosa	Primary Addison’s disease
Low body weight	Low body weight
Distorted perception of body image	Normal perception of body image
Fatigue	Fatigue and weakness
Low BMI	Low BMI
Restriction of energy intake	No restriction of energy intake; salt craving
Induced vomiting	Nausea, vomiting, abdominal pain and diarrhoea
Predominant malnutrition	Predominant dehydration
Hypotension and bradycardia	Hypotension and normal or increased heart rate
Hypothermia	Normothermic
Ammenorrhoea	Ammenorhoea less frequent (25%)
Decreased: GnRH, LH, FSH, IGF-1, testosterone, T3, T4, ADH	Electrolyte abnormalities: hyponatraemia, hyperkalemia
Increased: GH, cortisol	Decreased: cortisol; elevated: ACTH
Hypoglycaemia	Hypoglycaemia
Hyperpigmentation, xerosis	Hyperpigmentation of skin, mucosa, palmar creases, axillae, gingival borders
Lanugo body hair and hirsutism	Decreased pubic and axillary hair development in pubertal patients

ACTH, adrenocorticotropic hormone; ADH, antidiuretic hormone; BMI, body mass index; FSH, follicle-stimulating hormone; GH, growth hormone; GnRH, gonadotropin-releasing hormone; IGF-1, insulin-like growth factor 1; LH, luteinising hormone; T4, thyroxine; T3, triiodothyronine.

Once clinical suspicion of AD arises, the diagnosis is confirmed by low cortisol levels, elevated adrenocorticotropic hormone (ACTH), and the corticotropin stimulation test. However, in cases of adrenal crisis, treatment should not be delayed while awaiting test results. Immediate administration of corticosteroids is recommended [4]. To establish the etiology, adrenal autoantibodies or imaging tests could be necessary.

Psychiatric symptoms [7] may be the first manifestation of the disease and include depressive symptoms (low mood, asthenia, hypovolition, and tendency to isolate) or psychotic symptoms (delusions and auditory or visual hallucinations), though the latter are more commonly linked to other endocrine disorders. In the 2006 review by Anglin *et al*. [7], 25 cases were identified in the English literature where AD was associated with psychiatric symptoms. The most common psychiatric manifestations were delusions (56%), depression (44%) and hallucinations (40%). Interestingly, weight loss, nausea, and vomiting (symptoms that could lead to an initial misdiagnosis of an eating disorder) were not considered psychiatric symptoms in this review.

## Case Presentation

A 56-year-old woman was admitted to the Internal Medicine Department due to electrolite imbalances and asthenia. A psychiatric consultation was requested because the patient has already been admitted twice with symptoms of asthenia, vomiting, and weight loss. The medical team noted a complicated family situation involving a tumultuous relationship with her family, who were unable to manage her physical and emotional needs effectively.

The patient was under the care of a private psychiatrist, who recommended psychiatric admission once her physical condition stabilized, suspecting an eating disorder.

The patient’s medical history included a recent admission to the Digestive Department 10 days prior due to a year-long history of recurrent vomiting, which had led to worsening renal function, hyponatremia, hyperkalemia, and significant hypomagnesemia. During that admission, diagnostic tests including gastroscopy, ultrasound, and an abdomino-pelvic Computer Tomography scan were performed, but no organic cause was found for the vomiting, apart from evidence of chronic gastritis. Other tests, including antibodies for celiac disease, returned negative results. The patient was treated with intravenous fluids, proton pump inhibitors, and antiemetics as needed, leading to resolution of her symptoms, and she was subsequently discharged without further treatment.

The patient’s medical history also included hypothyroidism (treated with hormone replacement therapy), spondyloarthropathy with Human Leukocyte Antigen B27-negative bilateral sacroiliitis (managed by Rheumatology), and Janus Kinase-2 positive polycythemia. Her regular medications include Levothyroxine 112 mg daily, Bromazepam 1.5 mg every 12 hours, Duloxetine 60 mg daily, and Pantoprazole 40 mg daily.

On arrival at the emergency room, the patient presented with marked electrolyte abnormalities: hyponatremia (127 mEq/L), hypomagnesemia (1.23 mg/dL), and hyperkalemia (6.3 mEq/L), as well as metabolic acidosis and exacerbated chronic renal failure (serum creatinine 1.65 mg/dL). Blood pressure was 95/54 mmHg, and heart rate was 113 bpm. She reported a weight loss of 10 kg over the last year, with a current weight of 50 kg and a body mass index (BMI) of 18 kg/m^2^.

The patient acknowledged recurring vomiting for 18 months, with episodes occurring 2–3 times per week.

The patient categorically denied any intention to lose weight and described herself as being “skins and bones”, comparing herself to a person suffering from cancer. She also denied engaging in self-induced vomiting. She recognized a desire to lose weight in the past and a fear of regaining her maximum weight (65 kg), but attributed her inability to eat to lack of energy and stressful personal circumstances, expressing concern about the poor condition of her house, the death of a sibling and the stress she suffered at work.

The patient’s family reported significant self-neglect and abandonment of household chores over the past year, but they denied observing self-induced vomiting. They noted that she has restricted certain food groups after developing a hazelnut allergy a few months prior. The family observed that the patient sometimes skipped meals but attributed this behavior to her laziness and lack of energy. When questioned, some family members dismissed the possibility of their relative having an eating disorder (ED), though they did acknowledge that she might be suffering from a mental disorder.

A psychiatric consultation concluded that the patient did not meet the criteria for anorexia nervosa or bulimia due to the absence of self-induced vomiting and the atypical electrolyte imbalance, which did not match the usual patterns seen in bulimia or purging anorexia.

The differential diagnosis with a depressive disorder was also considered, as the patient presented marked anergy and avolition. However, this diagnosis was quickly ruled out for several reasons. First, the patient did not clearly report a low mood. On the other hand, the general clinical picture, with vomiting and electrolyte alterations, pointed more to a pathology of organic origin.

A subsequent consultation with the Endocrinology Department led to a suspected diagnosis of Addison’s disease based on her electrolyte abnormalities and skin hyperpigmentation. Given the patient’s history of autoimmune conditions, testing for anti-21-hydroxylase antibodies was performed and returned positive, confirming a diagnosis of autoimmune adrenalitis. Treatment with dexamethasone resulted in dramatic improvement, with normalization of electrolytes and clinical symptoms. In the last analysis prior to discharge, sodium (135 mEq/L), potassium (4.2 mEq/L), magnesium (1.7 mg/dL) and creatinine (1 mg/dL) were in range. In subsequent follow-up by Endocrinology and outpatient Psychiatry, the persistence of improvement was confirmed, making it possible to withdraw antidepressant treatment. In a subsequent consultation, the patient was definitively discharged from outpatient Psychiatry since the improvement in anergia, abulia, as well as a notable weight gain, persisted months after the withdrawal of antidepressant treatment. Ongoing Endocrinology follow-up was scheduled for lifelong corticosteroid replacement therapy.

In a recent contact, the patient referred to great overall improvement, showed no concern about her weight gain (65 kg, with a BMI of 23.6 kg/m^2^) and she reported no significant side effects.

**Fig. 1.**
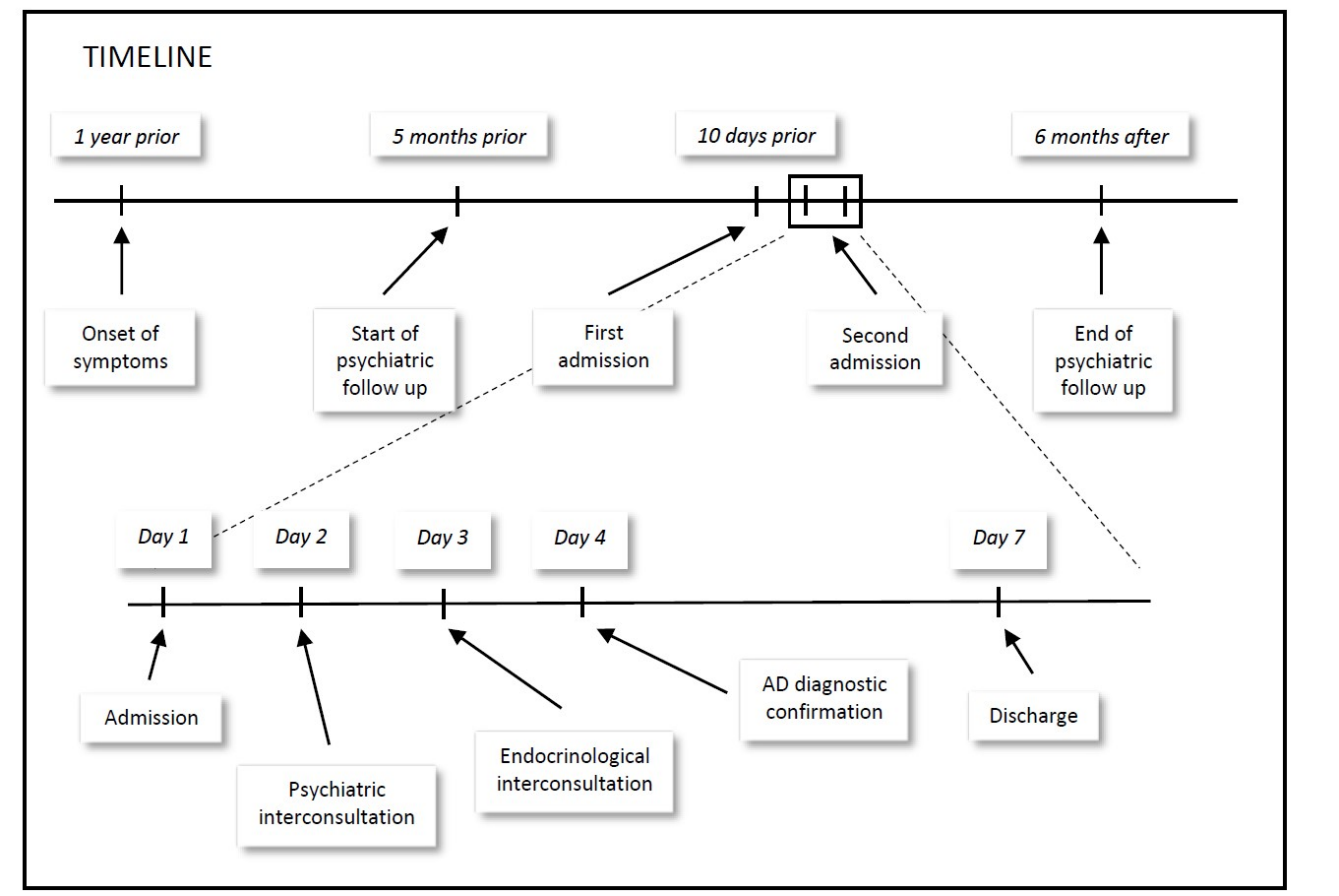
**Timeline**.

The patient’s evolution has been recorded on a timeline (Fig. 1).

This case presentation follows the CARE checklist, which is available in the supplementary material under the name “**Supplementary file 1**”.

## Literature Review

A search in PubMed under the terms “(((eating disorder) OR (bulimia)) OR (anorexia nervosa)) AND (addison)” yielded 35 results. Of these, 11 (9 in English and 2 in German) described patients who were initially diagnosed with an eating disorder, only to later receive a diagnosis of Addison’s disease. In this review, only English-language publications were included to facilitate data analysis.

Data from these 10 cases (9 published in English and the present case) were analysed and summarized in Table 2 (Ref. [6, 8, 9, 10, 11, 12, 13, 14, 15]).

**Table 2.** **Demographic and clinical data of reviewed cases**.

ID	Gender*	Age	Hyperpigmentation	Potassium (mEq/L)	Sodium (mEq/L)	Hypotension	BMI** (Kg/m^2^)	Diagnostic delay*** (months)
Adams *et al*. [8]	F	18	YES	4.3	127	YES	No data	6
Blaustein *et al*. [9]	F	12	YES	5.2	124	YES	9.2	No data
Feeney and Buell [10]	F	22	YES	4.8	136	YES	14.5	12
Keljo and Squires [11]	F	15	YES	3.9	134	YES	16.6	7
Lazare [12]	F	30	YES	4.8	126	YES	17	12
Morais *et al*. [13]	F	13	YES	6.4	117	YES	13.8	12
Nicholls *et al*. [6]	F	15	YES	4.2	129	YES	12.7	6
Nichols *et al*. [14]	F	14	YES	5.2	132	YES	15.2	No data
Tobin and Morris [15]	M	20	NO	3.8	126	YES	14.3	6
Present case	F	56	YES	6.3	127	YES	18	12

*Gender: Male (M)/Female (F); **BMI, body mass index; ***Diagnostic delay: time from the onset of weight loss until the diagnosis of Addison’s disease. Note: In some of the articles, the notation “mmol/L” was used instead of “mEq/L”. Since, in the case of Sodium and Potassium, 1 mmol/L = 1 mEq/L, for greater clarity only the last one has been used in this review.

## Discussion

Regarding the analysis of the obtained data, the distribution of the sample by age and sex overlaps with the current prevalence observed for eating disorders (ED) in our environment [2, 3]. It is striking that hyperpigmentation as a clinical sign was absent in only one of the cases. Hyponatremia also seems almost universal, with only one case not being affected. However, hyperkalemia seems to be a rarer finding, and, in this sample, it is only present in 40% of cases. On the other hand, hypotension and malnutrition, in some cases severe, are very frequent findings as reported in this series, with a significant weight loss (>10% of the usual weight in the last year) in all cases that provide data.

8 of the 10 patients were diagnosed with ED before receiving the diagnosis of Addison’s disease (AD). In the remaining 2 cases, an ED was considered as a potential diagnosis; however, in one case, the psychiatric evaluation ruled out an ED, and in the other, AD was diagnosed before the suspicion of an ED was fully explored.

Data were collected regarding the delay in the diagnosis of AD, specifically measuring the time from the onset of weight loss. Weight loss was chosen as the starting point because it is a common symptom in both conditions and was present in all the cases reviewed. The analysis revealed that, in all cases where data were available, the diagnostic delay ranged from 6 to 12 months.

## Conclusions

The analysis of this case series reveals that AD is often misdiagnosed as an ED due to overlapping symptoms such as weight loss, vomiting, and fatigue. However, clinical features like hyperpigmentation, electrolyte imbalances, and hypotension are key differentiators for AD. This case highlights the importance of considering AD in the differential diagnosis of patients presenting with weight loss, vomiting, and psychiatric symptoms, particularly when there is a lack of typical ED behaviours such as self-induced vomiting. This becomes especially important when you consider that early recognition and treatment of AD can dramatically improve outcomes. Therefore, it is essential that Mental Health services, particularly those treating EDs, work closely with Endocrinology services to ensure accurate diagnoses.

Last but not least, we should try to see the patient as a whole, instead of focusing only on aspects of our specialty. For example, in the case just presented, we would ask whether a psychiatric diagnosis explains everything that happens to the patient and, if not, whether there is some medical entity that can explain all of his symptoms. In this case, it seems clear that the symptoms that the patient presented and that led her to go to the emergency room were better explained by AD than by any other cause. The outcome of the clinical follow-up strengthens this thesis since the symptoms that pointed to a psychiatric disorder (vomiting, weight loss, avolition and anergy) disappeared with the introduction of endocrine treatment.

A key aspect of this approach is the importance of ruling out non-psychiatric conditions when psychiatric symptoms first appear. This step is critical to avoid misdiagnosis and ensure that underlying medical conditions are not overlooked. Additionally, it helps to prevent the introduction of subjective opinions that could be stigmatizing to the patient. Ensuring a thorough, unbiased evaluation not only safeguards the patient’s well-being but also promotes a more compassionate and accurate diagnosis.

The value of publishing such cases lies in raising awareness among professionals who diagnose and treat EDs, such as psychiatrists and psychologists, encouraging them to consider AD in their differential diagnoses. Additionally, it is important to inform Family Medicine and Pediatrics professionals, as they are often the first to diagnose suspected ED or AD. We address specifically Pediatricians since more than half of the cases published in the literature are of under-age patients.

From this point of view, a criticism that can be made of this article is, precisely, that it is published in a journal aimed at specialists in Psychiatry. However, we hope that publications like this one will help to place greater emphasis on the differential diagnosis with psychiatric disorders and, especially with ED, when reviews on AD, aimed at general practitioners, are published.

## Availability of Data and Materials

The datasets used and/or analyzed during the current study are available from the corresponding author on reasonable request.

## Supplementary Material

Supplementary material associated with this article can be found, in the online version, at https://doi.org/10.62641/aep.v53i3.1840.
